# Neuroprotective Effect of Ginseng Fibrous Root Enzymatic Hydrolysate against Oxidative Stress

**DOI:** 10.3390/molecules27227824

**Published:** 2022-11-13

**Authors:** Yuhua Zhu, Ziyan Wang, Shuxuan Yu, Chong Zhao, Baofeng Xu, Rui Liu, Li Xu, Yi Guo

**Affiliations:** 1 Key Laboratory for Molecular Enzymology and Engineering, The Ministry of Education, National Engineering Laboratory for AIDS Vaccine, School of Life Sciences, Jilin University, Changchun 130012, China; 2 Department of Neurosurgery, First Hospital of Jilin University, Changchun 130021, China; 3 Department of VIP Unit, China-Japan Union Hospital of Jilin University, Changchun 130033, China

**Keywords:** oxidative stress, ginseng, antioxidants, Alzheimer’s disease, neuroprotection

## Abstract

Oxidative stress is one of the potential causes of nervous system disease. Ginseng extract possesses excellent antioxidant activity; however, little research on the function of the ginseng fibrous root. This study aimed to investigate the neuroprotective effects of ginseng fibrous root to alleviate the pathogenesis of Alzheimer’s disease (AD) against oxidative stress. Ginseng fibrous root enzymatic hydrolysate (GFREH) was first prepared by digesting ginseng fibrous roots with alkaline protease. In vitro, the GFREH showed antioxidant activities in free radical scavenging mechanisms. With a cellular model of AD, GFREH inhibited the increase in Ca^2+^ levels and intracellular ROS content, maintained the balance of mitochondrial membrane potential, and relieved L-glutamic acid-induced neurotoxicity. In vivo, GFREH improved the survival rate of *Caenorhabditis elegans* (*C. elegans*) under oxidative stress, upregulated SOD-3 expression, and reduced reactive oxygen species (ROS) content. Therefore, our findings provide evidence for the alleviation effect of GFREH against oxidative stress in neuroprotection, which may accelerate the development of anti-Alzheimer’s drugs and treatments in the future.

## 1. Introduction

Oxidative stress (OS) refers to an imbalance between oxidation and antioxidation in the body, leading to the infiltration of neutrophils, increased secretion of proteases, and production of large amounts of oxidative intermediate products. It is mediated by a dynamic imbalance in the production and consumption of free radicals [1]. Reactive oxygen species (ROS) contain superoxide anions, hydroxyl free radicals, and hydrogen peroxide [2]. ROSs have a dual role in the body, wherein small amounts of free radicals or ROSs produced by organisms play an important role in normal physiological regulation such as host defense, gene transcription, and programmed cell death. However, once ROS levels are excessive, OS is generated, leading to cell damage, lipid peroxidation, nucleic acid damage, and ultimately various diseases [3,4,5,6]. OS has been recognized as an important factor in the development of cardiovascular diseases [7], neurodegenerative diseases [8], cancer [9], and other diseases [10]. Because neurons require large amounts of oxygen but low levels of antioxidants, the nervous system is particularly sensitive to oxidative damage caused by OS, which is one of the primary causes of Alzheimer’s disease (AD) [11]. Human neuroblastoma SH-SY5Y cells are widely used to study the pathogenesis of AD caused by OS [12]. Drugs currently in clinical use have toxic side effects and only alleviate the symptoms of AD but do not cure the disease. Therefore, safe and effective natural compounds are used as a means of treatment or adjunctive treatment of AD. A large number of studies have shown that the neuroprotective effects of plant extracts are associated with antioxidant properties [13,14]. Research on natural extracts and their biological activities is currently a hot topic in pharmaceuticals and health food. Many efforts are devoted to natural compounds, especially those with antioxidant, anti-inflammatory, neuroprotective, and immune-stimulating properties [15].

Ginseng, belonging to the genus *Panax* and family Araliaceae, is a famous and precious medicinal material, widely used as a traditional Chinese medicine and health food in many countries [16]. Ginseng contains a variety of biologically active ingredients including ginsenosides, polysaccharides, volatile oils, polyacetylene, peptides, fatty acids, and alkaloids [17,18,19]. These pharmacological compounds show various antioxidant [20], anti-inflammatory [21], antidiabetic [22], antibacterial [23], anticardiovascular disease [24], and antineurodegenerative activity [25]. Many studies have shown that ginseng extract has neuroprotective effects, can improve symptoms, and hinder AD progression in patients by reducing Aβ deposition and tau hyperphosphorylation [26]. The combined use of ginseng and drugs can reduce toxicity and improve therapeutic effects, indicating that ginseng can be used as an auxiliary means for the treatment of AD. Ginseng fibrous roots are the finely branched roots of ginseng, which have certain biological activity and lower costs, compared with that of ginseng. Therefore, the study on ginseng fibrous roots expands the medicinal value of ginseng.

*Caenorhabditis elegans* (*C. elegans*) is a type of eukaryote and model organism for studying various physiological functions [27]. At present, *C. elegans* is often used for research on toxicity, lipid and carbohydrate metabolism, neurodegenerative diseases, and immune response [28,29,30,31]. As a model organism, *C. elegans* has the following advantages: short life cycle, fast reproduction, low feeding cost, complete genome sequencing, transparent body, convenient observation of the expression of multiple fluorescent proteins, and conservation of many genes and signal pathways between *C. elegans* and humans [32]. Many researchers have used *C. elegans* to study oxidative stress induced disease. For example, Liu et al. have demonstrated that carnitine promoted the recovery of paraquat or juglone -induced oxidative stress in *C. elegans* and decreased the toxic reaction induced by human amyloid (Aβ) [33].

**Figure 1:**
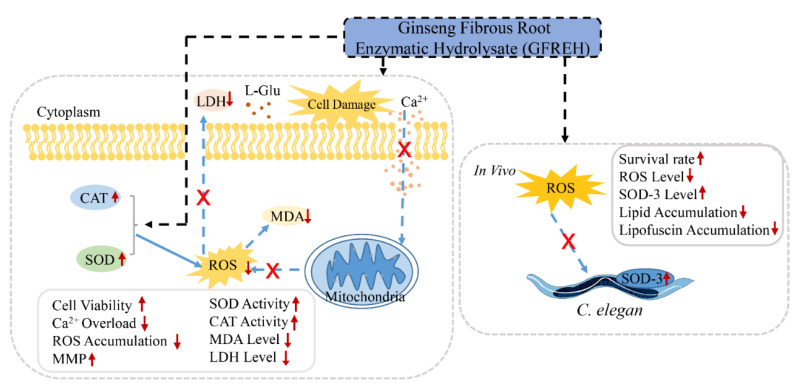
Schematic experiments of GFREH’s effect on oxidative stress and neurotoxicity. In cell assays, AD model was established by treating L-Glu. GFREH administration increased cell viability, which may be achieved by reversing intracellular Ca^2+^ influx, maintaining MMP stability, and upregulating the expression of antioxidant enzymes to eliminate intracellular ROS. In vivo, GFREH treatment increased nematodes’ survival rate under OS conditions, upregulated the expression of SOD-3, and decreased intracellular ROS in nematodes exposed to juglone. GFREH treatment also reduced lipid and lipofuscin accumulation. (GFREH: ginseng fibrous root enzymatic hydrolysate; OS: oxidative stress; L-Glu: L-glutamic acid; ROS: reactive oxygen species; MMP: mitochondrial membrane potential; SOD: superoxide dismutase; CAT: catalase; LDH: lactate dehydrogenase; MDA: Malondialdehyde).

In this study, ginseng fibrous root enzymatic hydrolysate (GFREH) was first prepared by digesting ginseng fibrous roots with alkaline protease. The SH-SY5Y cell was used to explore the antioxidative effect of GFREH against oxidative stress. In vivo, the neuroprotective role was evaluated in *C. elegans* by improvement of the antioxidant system, which is relevant for assessing the biological activity of GFREH in the human body (Figure 1).

## 2. Results

### 2.1. GFREH Effectively Scavenged Free Radicals In Vitro

**Figure 2:**
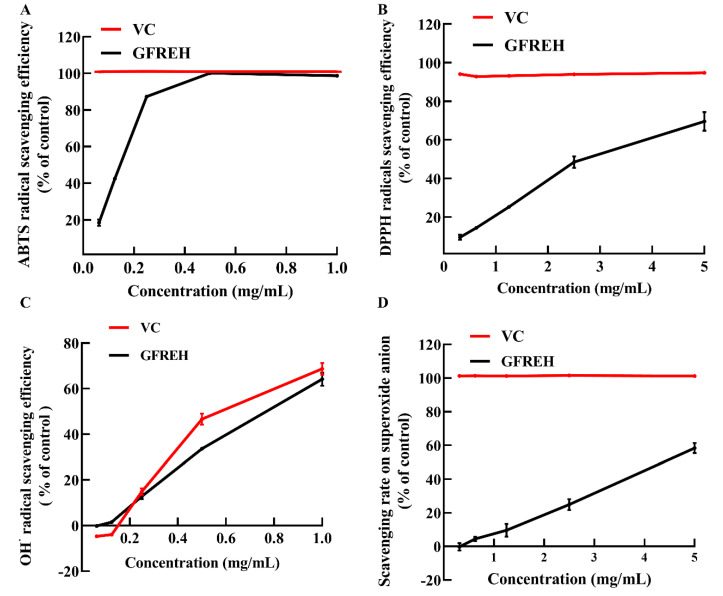
The role of GFREH in the scavenging of free radicals. (**A**) GFREH effectively scavenged ABTS free radicals in vitro (n = 3). (**B**) GFREH greatly scavenged DPPH free radicals in vitro (n = 3). (**C**) GFREH effectively scavenged OH∙ free radicals in vitro (n = 3). (**D**) GFREH effectively scavenged superoxide anion in vitro (n = 3).

We evaluated the free radical-scavenging ability of GFREH using vitamin C (VC) as a positive control to explore its antioxidant activity in vitro. As shown in Figure 2A, GFREH increased 2’-azino-bis (3-ethylbenzothiazoline-6-sulfonic acid) (ABTS) free radical-scavenging activity in a concentration-dependent manner, and the scavenging ratio was the same as that of VC, reaching 100% at a concentration of 0.5 mg/mL. Likewise, GFREH improved 2-diphenyl-1-picrylhydrazyl (DPPH) free radical-scavenging activity (Figure 2B), and the scavenging rate was 69.55%. Figure 2C showed the ability of GFREH to eliminate hydroxyl radicals. The results showed that both VC and GFREH increased the scavenging rates of hydroxyl radicals. The concentration of both was 1 mg/mL, and the scavenging rates reached 68.7% and 64.1%, respectively. In Figure 2D, the scavenging rate of GFREH for superoxide anions was 58.41%.

### 2.2. GFREH Protected SH-SY5Y Cells from L-Glu-Induced Apoptosis

**Figure 3:**
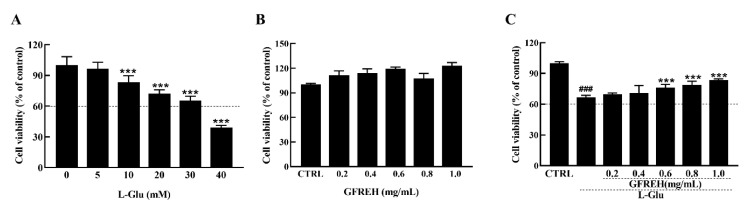
(**A**) The effect of L-Glu on apoptosis of SH-SY5Y cells, (n = 6). (**B**) GFREH treatment alone for 24 h caused no decrease in the viability of SH-SY5Y cells, (n = 6). (**C**) GFREH pretreatment for 4 h and incubation with L-Glu for an additional 24 h improved cell viability, (n = 6). The data are presented as the mean value ± SD. ### *p* < 0.001 vs. CTRL, *** *p* < 0.001 vs. L-Glu-treated cells. CTRL: untreated cells, L-Glu, cells treated with L-Glu.

The MTT (3-(4, 5-dimethylthiazol-2-yl)-2, 5-diphenyltetrazolium Bromide) assay (Biotechnology, Shanghai, China) was used to analyze the viability of the SH-SY5Y cells treated with different concentrations of L-Glu for 24 h. Data were collected by a microplate reader (Infinite F200 Pro; Tecan Group AG, Männedorf, Switzerland). Exposure to L-Glu for 24 h resulted in apoptosis, and the cell viabilities decreased with increased concentrations (Figure 3A). Treatment of the SH-SY5Y cells with 30 mM L-Glu caused 35% cell death. GFREH alone had no toxic effect on SH-SY5Y cells and promoted cell proliferation (Figure 3B). Moreover, a 4 h preincubation and coincubation with L-Glu improved cell viability by 20% compared with L-Glu-treated SH-SY5Y cells (Figure 3C).

### 2.3. GFREH Reversed Ca^2+^ Overload

**Figure 4:**
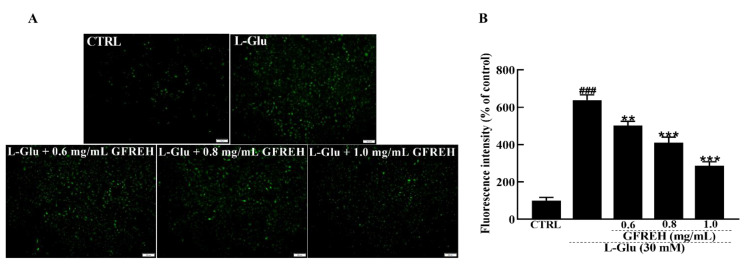
Fluorescence microscopy images of intracellular calcium levels. (**A**) Intracellular Ca^2+^ overload caused by L-Glu was reversed by GFREH pretreatment, as detected by Fluo-4-AM staining. The scale bar is 100 μm. (**B**) Fluorescence quantification by ImageJ software (version: 1.51), (n = 3). The data are presented as the mean value ± SD. ### *p* < 0.001 vs. CTRL, ** *p* < 0.01, *** *p* < 0.001 vs. L-Glu-treated cells.

Abnormal intracellular free Ca^2+^ concentrations play an important role in the progression of cell death. Fluo-4-AM staining was used to measure the concentration of free intracellular Ca^2+^. Compared with the CTRL group, treatment of SH-SY5Y cells with 30 mM L-Glu for 24 h resulted in an overload of intracellular calcium ions, as indicated by green fluorescence. After treatment with GFREH (e.g., 0, 0.6, 0.8, and 1.0 mg/mL), the green fluorescence was significantly decreased (Figure 4), indicating its suppressive effect on intracellular calcium overload.

### 2.4. GFREH Protected Mitochondrial Function

**Figure 5:**
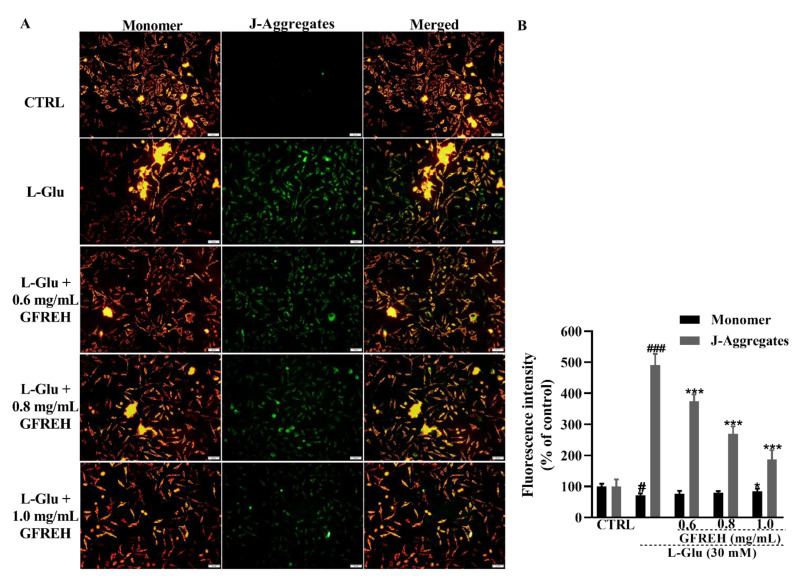
(**A**) The effect of MMP caused by 24 h L-Glu exposure was strongly restored by 4 h GFREH pretreatment, as analyzed by JC-1. Scale bar is 100 μm. (**B**) Fluorescence quantification by ImageJ software (version: 1.51), (n = 3). The data are presented as the mean value ± SD. ^#^ *p* < 0.01 and ^###^ *p* < 0.001 vs. CTRL, * *p* < 0.01 and *** *p* < 0.001 vs. L-Glu-treated cells.

Mitochondrial function was measured using JC-1 staining. A decrease in MMP is a hallmark of apoptosis. JC-1 was present in the mitochondrial matrix of cells with higher MMP as a polymer, producing red fluorescence, whereas it was present in apoptotic cells with decreased MMP in the form of monomers, producing green fluorescence. We observed that green fluorescence accumulated in cells treated with L-Glu, and this accumulation could be removed by treating cells with GFREH (Figure 5). GFREH pretreatment for 4 h, followed by incubation with L-Glu for an additional 24 h, significantly decreased the intensity of green fluorescence in JC-1 staining, indicating its protective effect on mitochondrial function.

### 2.5. GFREH Reduced ROS Accumulations

**Figure 6:**
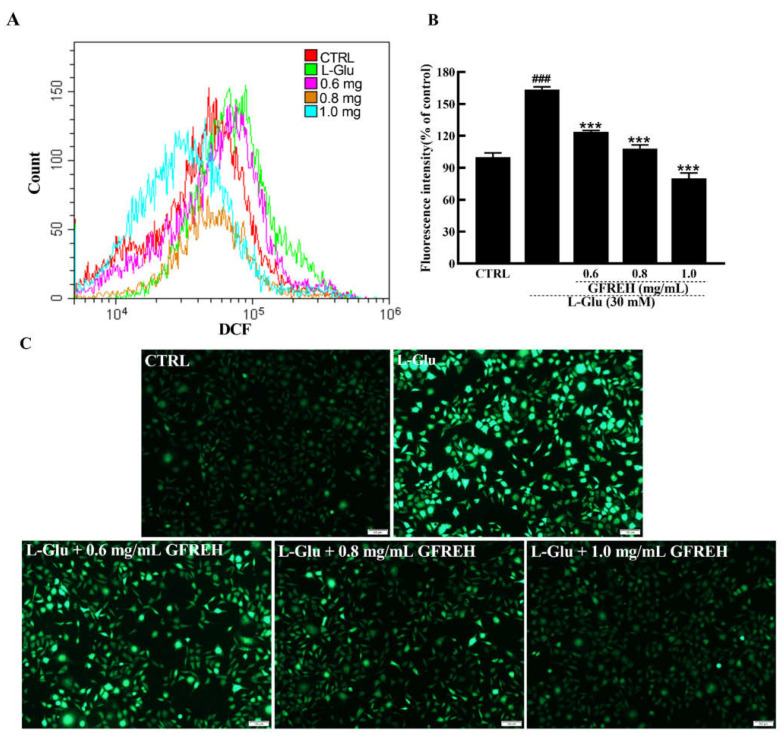
ROS are detected by DCFH-DA, which produces green fluorescence in the presence of ROS. (**A**) ROS contents in SH-SY5Y cells detected by a flow cytometer. (**B**) Cartogram of ROS in SH-SY5Y cells under different treatments, (n = 3). The data are expressed as the mean value ± SD. ^###^ *p* < 0.001 vs. CTRL, *** *p* < 0.001 vs. L-Glu-treated cells. (**C**) Pictures of SH-SY5Y cells are imaged by fluorescence microscopy. Scale bar is 100 μm.

ROS overproduction eventually leads to cell dysfunction and apoptosis. ROS production in SH-SY5Y cells was also measured. As shown in Figure 6, the intracellular ROS levels were detected by flow cytometer, and the green fluorescence intensities of SH-SY5Y cells activated by L-Glu were significantly higher than those of cells in the medium, whereas the fluorescence intensity decreased after treatment with different GFREH concentrations. After treatment with 0.6, 0.8, and 1.0 mg/mL GFREH, the ROS scavenging efficiency was 39.42%, 55.35%, and 83.32%, respectively, suggesting its inhibition of ROS accumulation, which showed a similar trend as that seen in images obtained with the fluorescence microscope.

### 2.6. Effect of GFREH on Antioxidative Enzymes in Cells

**Figure 7:**
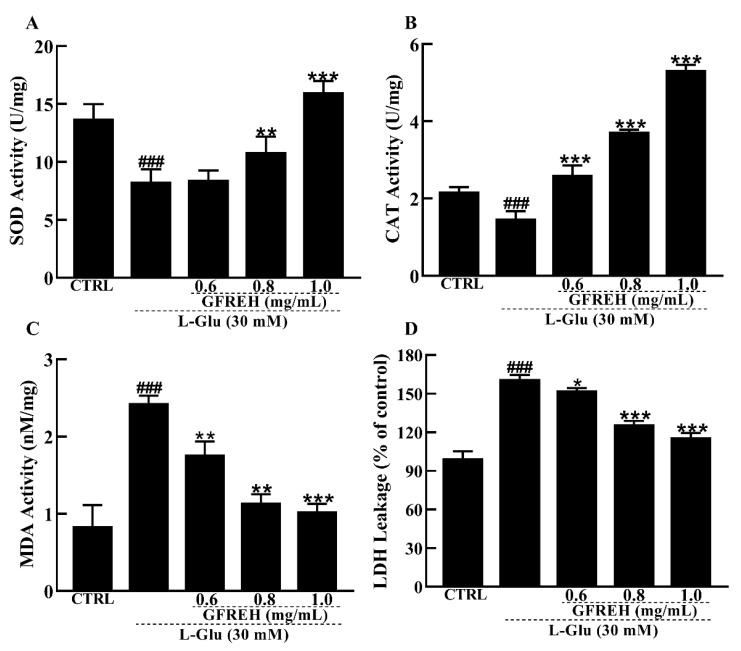
Effect of GFREH antioxidative enzymes in SH-SY5Y cells. The (**A**) SOD activity and (**B**) CAT activity induced by L-Glu were significantly increased by pretreatment with GFREH. The (**C**) MDA levels and (**D**) LDH release levels induced by L-Glu were significantly decreased by pretreatment with GFREH (n = 5). The data are presented as the mean value ± SD. ^###^ *p* < 0.001 vs. CTRL, * *p* < 0.05, ** *p* < 0.01, *** *p* < 0.001 vs. L-Glu-treated cells.

The antioxidant enzyme system is one of the main ways that cells can scavenge ROS in response to oxidative stress. Therefore, we measured the activities and levels of antioxidant enzymes. Our data showed that SOD and CAT activities were significantly lower in the AD cell model group than in the control group, while the SOD and CAT activities were significantly increased in the pretreatment group with GFREH (Figure 7A,B). In addition, the MDA and LDH leakage levels were reduced by GFREH pretreatment (Figure 7C,D).

### 2.7. Survival Analysis under Stress Conditions in C. elegans

**Figure 8:**
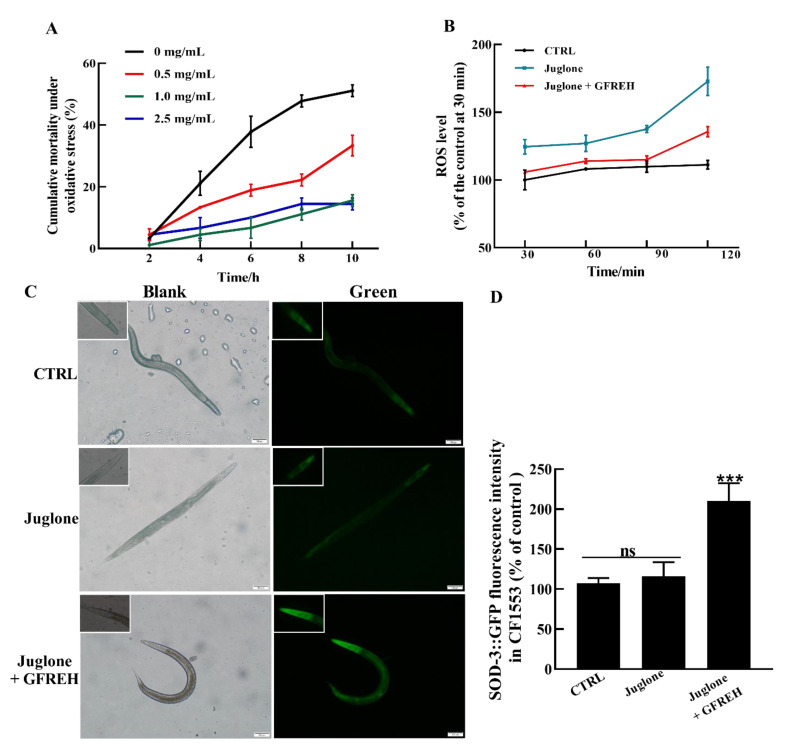
The protective effect of GFREH on *C. elegans* under stress conditions. (**A**) Different concentrations of GFREH reduced the cumulative mortality of *C. elegans* under oxidative stress, and the optimal concentration was 1 mg/mL, (n = 30). (**B**) GFREH reduced the ROS level in *C. elegans*, (n = 30). (**C**) The picture of *C. elegans* imaged by fluorescence microscopy. (**D**) GFREH upregulated the expression of SOD-3 in nematodes, (n = 30). Scale bar is 100 μm. The data in A, B, and D are presented as the mean value ± SD. *** *p* < 0.001 vs. juglone group. CTRL: untreated worms, Juglone: juglone-treated worms, Juglone + GFREH: Juglone, and 1 mg/mL GFREH-treated group.

Further experiments were conducted to evaluate the effects on OS in vivo. The results revealed that GFREH significantly reduced nematode mortality under OS conditions (Figure 8A). The ROS levels in worms were then measured. Compared with the control group, the ROS level of worms exposed to juglone was obviously increased. After the administration of 1.0 mg/mL of GFREH, the accumulation of ROS in the juglone-induced worms was inhibited, indicating that ROS in nematodes could be removed by GFREH (Figure 8B). To further investigate the protective mechanism of GFREH in *C. elegans*, the expression of the *sod-3* gene in transgenic *C. elegans* CF1553 was analyzed. As shown in Figure 8C,D, the expression of SOD-3 protein was upregulated after treatment with GFREH.

### 2.8. GFREH Improved the Health of C. elegans

**Figure 9:**
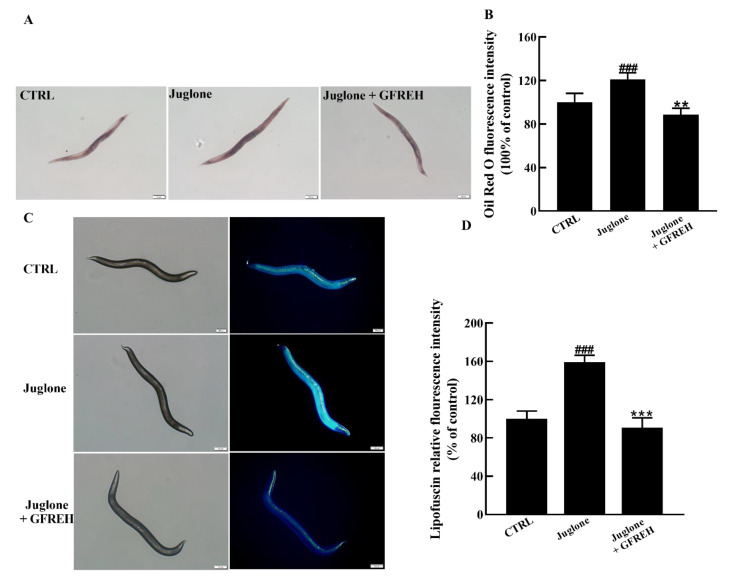
*C. elegans* N2 pictures imaged by fluorescence microscope. (**A**) Oil red O staining image of lipid accumulation in *C. elegans* N2 and (**B**) quantitation of the fluorescence by ImageJ software (version: 1.51) (n = 6). (**C**) Autofluorescence image of lipofuscin accumulation in *C. elegans* N2 and (**D**) quantitation of the fluorescence by ImageJ software (version: 1.51) (n = 6). Scale bar is 100 μm. The data in B and D are presented as the mean value ± SD. ^###^ *p* < 0.001 vs. CTRL, ** *p* < 0.01 and *** *p* < 0.001 vs. the Juglone group.

The accumulation of lipids and lipofuscin in *C. elegans* is an important indicator of health. Therefore, we verified the effect of GFREH on the accumulation of lipids and lipofuscin in worms. Compared with the control group, the contents of lipids and lipofuscin significantly increased in the juglone-induced group. After administering 1 mg/mL GFREH to the juglone-induced worms, the accumulation of lipid and lipofuscin was reduced. After calculation, the reduction rates of GFREH on lipid and lipofuscin were 32.48% and 68.24%, respectively (Figure 9A–D). The motor function of *C. elegans* also diminishes with age, and the rate of movement in solid media or liquid is also an indicator of its health.

## 3. Discussion

The development of natural products may meet the urgent needs of modern society for effective antineurodegenerative diseases [34]. The present study confirmed the antioxidative activities and neuroprotective properties of GFREH. They improved cell viability, inhibited Ca^2+^ overload, and upregulated the expression of antioxidant enzymes in vitro. Moreover, the administration of GFREH improved survival ability, increased SOD-3 expression, and reduced the ROS level in juglone-induced worms.

Since ancient times, ginseng has been used as a traditional medicine and functional food to prevent and treat a variety of diseases due to its multiple active ingredients [35]. However, the pharmacological action and molecular mechanism of ginseng root whiskers are still unclear. We verified that GFREH effectively scavenged ABTS free radicals, DPPH free radicals, OH**^∙^** free radicals, and superoxide anions in vitro. In response to the juglone that produces ROS, worms activate the expression of antioxidative genes to alleviate oxidative damage [33]. In this research, based on the juglone-induced oxidative damage model, we demonstrated that GFREH at a concentration of 1 mg/mL significantly improved the survival rate of nematodes under oxidative stress. Compared with the group exposed to juglone, the GFREH treatment group showed significantly reduced ROS levels and increased protein expression levels of SOD-3 in the worms. These results indicated that GFREH has good antioxidant effects both in vitro and in vivo.

OS and glutamate neurotoxicity are potential causes of Alzheimer’s disease [36]. Therefore, inhibiting neurotoxicity and removing excess ROS is also one of the means to prevent and treat Alzheimer’s disease. Glutamate is an endogenous excitatory neurotransmitter, mainly found in the cerebral cortex and hippocampus, and plays an important role in the neurotransmission, development, and synaptic plasticity of the nervous system [37]. However, excessive release of this excitatory agonist leads to neuronal dysfunction and is an important underlying cause of various neurodegenerative diseases [38]. Glutamate-induced neuronal damage is often accompanied by Ca^2+^ influx, resulting in impaired mitochondrial function and increased levels of ROS [39]. We found that the levels of intracellular Ca^2+^ were significantly increased and MMP levels were decreased after L-Glu treatment in SH-SY5Y cells, but these phenomena were reversed after exposure to GFREH. Dysregulation of oxidative signaling can cause or accelerate a range of pathological conditions [32]. The human body is equipped with two antioxidant systems, one of which is an enzyme antioxidant represented by SOD, CAT, and glutathione peroxidase [40]. SOD, CAT, and glutathione peroxidase catalyze the disproportionation of superoxide anion into H_2_O_2_, which is further reduced to oxygen and water [41]. The results of this study showed that GFREH exerted its neuroprotective activity via ROS inhibition and increased SOD and CAT activity. ROS attacks the cell membrane, causing damage to the cell membrane and leading to lipid peroxidation [42,43]. It has been shown that ent-Kaur-15-en-17-al-18-oic acid inhibits ROS and MDA production in Aβ-induced oxidative stress in neural cell models. Our results exhibited that MDA and LDH levels were obviously decreased after GFREH treatment, compared with the L-Glu treatment group. These results indicate that the GFREH protected the SH-SY5Y cells from L-Glu-induced oxidative damage. Our study provided new insights into the applications of ginseng as a potential neuroprotective agent to protect the nervous system and alleviate the effects of neurological diseases.

However, there are still some shortcomings in this study, which need to be improved in future work. First, GFREH is a mixture. It is best to perform further isolation and purification to prepare a single active ingredient, which facilitates mechanistic studies and target identification. Second, there are various glutamate receptors in the nervous system. One is ionotropic GluRs that are coupled with ion channels to form receptor channel complexes and mediate fast signal transmission, while the other is the metabotropic glutamate receptors that lead to metabolic changes through G-protein coupling, regulating the production of second messengers in cells [44]. Whether the mechanism by which GFREH inhibits neurotoxicity is related to glutamate receptors remains to be further investigated. This study shows new evidence for ginseng as an adjunctive treatment for Alzheimer’s disease and provides a basis for the development of anti-Alzheimer’s disease drugs.

## 4. Materials and Methods

### 4.1. Materials

Ginseng fibrous roots were collected from Changbai Mountain in Northeast China. Roots were crushed into powder using a pulverizer. Alcalase was purchased from Novo Nordisk (Shenyang Biochemical Processing Co. Ltd., Shenyang, China).

### 4.2. Preparation of GFREH

Ten grams of ginseng powder and 100 mL of distilled water were added to a 250 mL conical flask. The flask was incubated in a 50 °C water bath for 5 min, and the pH of the solution was adjusted to 8.0. The enzymatic hydrolysis reaction was initiated when the enzyme was added to the mixture. During the hydrolysis process, the pH of the solution was adjusted to 8.0 again, and the amount of alkali added was recorded. After hydrolysis, the enzyme was inactivated by boiling for 10 min, and the mixture was cooled to 25 °C and centrifuged at 8000× *g* for 15 min. The supernatant was then filtered, freeze dried, and stored at −20 °C. The degree of hydrolysis (DH) was determined using the pH stat method [45] 

### 4.3. In vitro Antioxidant Assay

#### 4.3.1. ABTS Radical-Scavenging Assay

The ABTS radical-scavenging activity of GFREH was determined according to the method described by Re et al., with some modifications [46]. ABTS (3.84 mg) and K_2_S_2_O_4_ (0.66 mg) were dissolved in ultrapure water and stored in the dark at room temperature for 12–16 h. Subsequently, the mixture was diluted with ethanol. The prepared sample solution (0.1 mL) and ABTS radical solution (0.9 mL) was added to the tube, mixed well, and incubated at room temperature in the dark for 20 min. The absorbances were measured at 734 nm using a spectrophotometer (UV-2700, Shimadzu (Suzhou) Instruments Manufacturing Co., Ltd., Suzhou, China). The sample solutions were replaced with ultrapure water and vitamin C (VC) in the blank control and the positive control. The ABTS radical-scavenging activity was calculated as follows:

$$\mathrm{ABTS}\;\mathrm{radical}\;\mathrm{scavenging}\;\mathrm{rate}\;\left(\%\right)=\left(A-A_{n}\right)/A\times 100$$

where *A* is the absorbance of the blank control and *A_n_* is the absorbance of the sample groups or positive groups. This experiment was performed in triplicate and the data are expressed as the mean ± standard deviation (SD).

#### 4.3.2. DPPH Radical-Scavenging Assay

The DPPH radical-scavenging activity of GFREH was determined according to the method described by Liu et al. with some modifications [47]. Different concentrations of aqueous sample solutions were prepared. The DPPH ethanol solution (0.5 mL of 0.2 mM) and sample solutions were mixed in equal volumes and incubated in the dark at room temperature for 30 min. The absorbance of each sample was measured at 517 nm using a spectrophotometer. The blank control and positive control were established using ethanol and VC, respectively, with equal volumes instead of the sample. The DPPH scavenging activity was calculated as follows:

$$\mathrm{DPPD}\;\mathrm{radical}\;\mathrm{scavenging}\;\mathrm{rate}(\%)=\left[1-\left(A_{i}-A_{0}\right)/A_{0}\;\right]\times 100$$

where *A_i_* is the absorbance of the sample groups or positive groups, and *A*_0_ is the absorbance of the control. This experiment was conducted in triplicate and the data are expressed as the mean ± SD.

#### 4.3.3. Hydroxyl Radical-Scavenging Assay

The hydroxyl radical-scavenging ability of GFREH was investigated in this study. Briefly, 0.14 mL of rhodamine B solution was mixed with 0.1 mL of the GFREH solution. Then, appropriate aliquots from the following solutions: 0.1 mL of 0.5 mM FeSO_4_, 0.1 mL of 20 mM H_2_O_2_, 0.2 mL of 0.01 mM Tris-HCl, and 0.36 mL of ultrapure water were added to the sample groups. Instead, ultrapure water was used in the hydroxyl radical test group, and the sample and H_2_O_2_ were used instead of ultrapure water in the blank control group. The hydroxyl radical-scavenging activity was calculated as follows:

$$\mathrm{Hydroxyl}\;\mathrm{radical}\;\mathrm{scavenging}\;\mathrm{rate}\;\left(\%\right)=\left(A_{i}-A_{0}\right)/(A-A_{0})\times 100$$

where *A_i_* is the absorbance of the sample groups or positive groups, *A* is the absorbance of the blank control, and *A*_0_ is the absorbance of the hydroxyl radical test group. This experiment was performed in triplicate and the data are expressed as mean ± SD.

#### 4.3.4. Superoxide Radical-Scavenging Assay

The superoxide radical-scavenging abilities were determined using the pyrogallol autoxidation method. Tris-HCl-EDTA solution (0.9 mL, pH 8.2), 0.1 mL of sample solution, and 2 µL of 45 mM were added to 1.5 mL Eppendorf tubes and mixed well, and the absorbance at 325 nm was measured quickly using a spectrophotometer. The first absorbance value was recorded at 1 min, and additional absorbance values were recorded every 30 s to provide a total of seven measurements over 4 min. The recording times and absorbance values were used as the abscissa and ordinate, respectively, to form a function graph, and the slope was defined as *K_i_*. Ultrapure water and VC were used as the blank control and positive control, respectively. The superoxide radical-scavenging ratio (%) was calculated using the following formula:

$$\mathrm{Superoxide}\;\mathrm{radical}\;\mathrm{scavenging}\;\mathrm{rate}\;\left(\%\right)=\left(K_{0}-K_{i}\right)/K_{0}\times 100$$

where *K*_0_ is the slope of the sample groups or positive groups, and *K_i_* is the slope of the blank group. This experiment was performed in triplicate and the data are expressed as the mean ± SD.

#### 4.3.5. Cell Culture

The human neuroblastoma cell line, SH-SY5Y (CRL-2266, ATCC, Manassas, VA, USA), was cultured to test the neuroprotective effects of GFREH against glutamate-mediated neurotoxicity. SH-SY5Y cells were grown in Dulbecco’s modified Eagle’s medium (Gibco-BRL, Grand Island, NY, USA) supplemented with 10% (*v*/*v*) fetal bovine serum (Kangyuan Biology, Tianjin, China), 100 U/mL penicillin, and 100 mg/mL streptomycin (Beijing Solarbio Science & Technology Beijing Co. Ltd., Beijing, China). Cells were incubated at 37 °C in a humid environment containing 5% CO_2_ and 95% air. Cells were subcultured at a ratio of 1:3 every two days.

#### 4.3.6. Cell Viability Assay

The MTT assay was used to evaluate the effect of GFREH on the viability of SH-SY5Y cells. SH-SY5Y cells were seeded into 96-well plates at a density of 5 × 10^3^ cells per well and grown overnight. The cells were treated with different concentrations of L-Glu (e.g., 0, 5, 10, 20, 30, and 40 mM) or GFREH (e.g., 0, 0.2, 0.4, 0.6, 0.8, and 1.0 mg/mL) for 24 h to determine the optimal doses. Then, the MTT solution (5 mg/mL) was added to each well and incubated for 4 h. Subsequently, the formazan crystals were dissolved in 150 µL DMSO, and the absorbance at 490 nm was measured using a microplate reader. After the optimal doses of L-Glu and GFREH were determined, the cells were pretreated with GFREH for 4 h and coincubated with 30 mM L-Glu for an additional 24 h. The MTT assay was then repeated as described above. All samples were evaluated in six parallels, and the experiment was repeated three times.

#### 4.3.7. Intracellular Ca^2+^ Concentration Assay

To analyze the intracellular Ca^2+^ concentration, Fluo-4-AM staining (Beyotime Biotechnology, Shanghai, China) was used. Cells were seeded into 6-well plates, treated with different GFREH concentrations (e.g., 0, 0.6, 0.8, and 1.0 mg/mL) for 4 h, incubated with 30 mM L-Glu for an additional 24 h and then loaded with Fluo-4-AM at a final concentration of 5 µM for 20 min at 37 °C in the dark. Afterward, the cells were washed three times with PBS and analyzed using a fluorescence microscope (IX73; Olympus, Tokyo, Japan).

#### 4.3.8. MMP Assay

The MMPs of SH-SY5Y cells were detected using a mitochondrial membrane potential assay kit with JC-1 (Beyotime Biotechnology, Shanghai, China). SH-SY5Y cells were plated into 6-well plates at a density of 2 × 10^5^ cells per well and incubated at 37 °C in a 5% CO_2_ incubator for 12 h. Cells were treated with different concentrations of GFREH (e.g., 0, 0.6, 0.8, and 1.0 mg/mL) for 4 h and then incubated with 30 mM L-Glu for 24 h. A JC-1 fluorescent probe was added to every well and incubated with cells for 20 min, followed by washing with phosphate buffer saline (PBS). The red and green fluorescence was measured using a fluorescent microscope (IX73; Olympus, Tokyo, Japan). The assay was evaluated in triplicate and repeated three times.

#### 4.3.9. ROS Level Assay

Intracellular ROS levels were detected using 2′, 7′-dichlorofluorescein diacetate (DCFH-DA) (Beyotime Biotechnology, Shanghai, China). Briefly, SH-SY5Y cells were seeded into 6-well plates at a density of 2 × 10^5^ cells per well and incubated at 37 °C in a 5% CO_2_ incubator for 12 h. Cells were then treated with different concentrations of GFREH (0, 0.6, 0.8, 1.0 mg/mL). After 4 h incubation, 30 mM of L-Glu was added and incubated for another 24 h. DCFH-DA solution was added to each well and incubated for 20 min. The intracellular green fluorescence intensity was detected using a fluorescent microscope (IX73; Olympus, Tokyo, Japan) and flow cytometer (Beckman Coulter, Brea, CA, USA). The assay was evaluated in triplicate and repeated three times.

#### 4.3.10. Antioxidant Enzyme Activity and Level Detection

Cells were seeded into 6-well plates and incubated as described in Section 4.3.9, and then the activities and levels were measured by SOD, CAT, MDA, and LDH assay kits (Beyotime Biotechnology, Shanghai, China). The assay was evaluated in five copies and repeated three times.

### 4.4. In Vivo Antioxidant Assay

#### 4.4.1. *C. elegans* Culture

Wild type *C. elegans* strain N2 and transgenic strain CF1553 were obtained from the Caenorhabditis Genetics Center (University of Minnesota, Minneapolis, MN, USA). Worms were cultured on nematode growth medium (NGM) agar plates at 20 °C and seeded with live *Escherichia coli* OP50 (*E. coli*) bacteria. L4 stage worms, which were generated by the synchronization of sodium hypochlorite, were used in all the experiments.

#### 4.4.2. ROS Assay and Intracellular ROS Level Detection in *C. elegans*

Worms were treated with 800 µM juglone and different GFREH concentrations (e.g., 0, 0.5, 1.0, 2.5 mg/mL) to determine the optimal dose of GFREH. After the optimal dose was determined, worms were kept on NGM plates with or without 1 mg/mL GFREH for two days and were then transferred onto plates with 1 mM juglone. Worm mortalities were recorded every two hours. Synchronized L4 worms were treated with 800 µM juglone at 20 °C for 2 h and then transferred onto plates with or without 1 mg/mL GFREH. The control group was not treated with juglone or GFREH. After two days of incubation, 30 worms were washed twice with PBS and incubated with 10 μM DCFH-DA in a black 96-well plate. The green fluorescence intensities were measured using a microplate reader with excitation at 488 nm and emission at 525 nm (Infinite F200 Pro; Tecan Group AG, Männedorf, Switzerland). The experiment was replicated three times independently.

#### 4.4.3. The Effect of GFREH on SOD-3∷GFP Expression in Transgenic *C. elegans* CF 1553 under OS

Worms that reached the late L4 stage were treated with 800 µM juglone for 2 h and then were transferred to plates with or without 1 mg/mL GFREH for 24 h. The control group was not treated with juglone or GFREH. To measure the expression levels of the SOD-3∷GFP in transgenic *C. elegans* treated with or without GFREH, the worms were paralyzed with 1% (*w*/*v*) levamisole and photographed using fluorescence microscopy (Olympus, Tokyo, Japan). Fluorescence intensities were detected using a microplate reader. The experiment was replicated three times independently.

#### 4.4.4. Detection of Lipid Accumulation in *C. elegans*

Thirty synchronized L4 stage (the final larval stage) animals in each group were treated with 800 µM juglone for 2 h and then transferred to plates with or without 1 mg/mL GFREH for 5 days. And the control group was not treated with juglone or GFREH. Nematodes were randomly selected and washed five times with PBS. After washing, worms were fixed with 1% paraformaldehyde for 20 min and washed with M9. Subsequently, the nematodes were repeatedly frozen and thawed at −80 °C and 40 °C three times and then dehydrated with a 60% isopropanol solution. Finally, the nematodes were stained using 60% Oil Red O for 30 min and washed three times with PBS. Intracellular lipid accumulation in *C. elegans* was analyzed using a fluorescence microscope and ImageJ software (version: 1.51; National Institutes of Health, Bethesda, MD, USA). The experiment was replicated three times independently.

#### 4.4.5. Detection of Lipofuscin Content in *C. elegans*

The treatment method was the same as the lipid accumulation experiment, but the administration time was extended to 10 days. Nematodes were anesthetized with 1% (*w*/*v*) levamisole, placed on a glass slide covered with 3% agar, and then photographed with a fluorescence microscope (IX73; Olympus, Tokyo, Japan). The experiment was replicated three times independently.

### 4.5. Statistical Analysis

All experimental results are expressed as the mean ± SD and were analyzed using GraphPad Prism 8.01 (GraphPad Software, San Diego, CA, USA). The statistical significance of the differences among the experimental groups, model groups, and control group were evaluated by one-way analysis of variance (ANOVA) followed by Tukey’s multiple comparisons test. (^#^ *p* < 0.05, ^##^ *p* < 0.01, ^###^ *p* < 0.001 vs. the CTRL group; * *p* < 0.05, ^**^ *p* < 0.01, *** *p* < 0.001 vs. the model group). All experiments were conducted in triplicate and replicated independently at least three times.

## 5. Conclusions

In conclusion, we prepared GFREH by enzymatic hydrolysis. GFREH has good antioxidant activity both in vitro and in vivo. In vitro, it efficiently scavenged free radicals, and in nematodes, it improved the survival rate of nematodes under OS conditions, up-regulated the expression of SOD-3, and eliminated ROS. GFREH inhibits L-Glu-induced neurotoxicity, which may be achieved by reversing intracellular Ca^2+^ influx and maintaining MMP stability. GFREH upregulated the expression of antioxidant enzymes to eliminate intracellular ROS and ultimately reduced L-Glu-induced oxidative damage in SH-SY5Y cells, implying that GFREH may serve as a neuroprotective agent to improve the treatment of nervous system disease.

## Data Availability

All data can be found in the manuscript.
